# Synthesis, characterization, crystal structure and supra­molecularity of ethyl (*E*)-2-cyano-3-(3-methyl­thio­phen-2-yl)acrylate and a new polymorph of ethyl (*E*)-2-cyano-3-(thio­phen-2-yl)acrylate

**DOI:** 10.1107/S2056989019011435

**Published:** 2019-08-23

**Authors:** Mahmoud Al-Refai, Basem F. Ali, Ala’a B. Said, Armin Geyer, Michael Marsch, Klaus Harms

**Affiliations:** a Department of Chemistry, Al al-Bayt University, Mafraq 25113, Jordan; b Faculty of Chemistry, Philipps University Marburg, Hans-Meerwein-Strasse 4, 35032 Marburg, Germany

**Keywords:** crystal structure, thio­phene-based cyano­acrylates, polymorph, crystal supra­molecularity

## Abstract

The synthesis, crystal structure and structural motif of two thio­phene-based cyano­acrylate derivatives, namely, ethyl (*E*)-2-cyano-3-(3-methyl­thio­phen-2-yl)acrylate (**1**), C_11_H_11_NO_2_S, and ethyl (*E*)-2-cyano-3-(thio­phen-2-yl)acrylate (**2**), C_10_H_9_NO_2_S, are reported. Derivative **1** crystallized with two independent molecules in the asymmetric unit, and derivative **2** represents a new monoclinic (*C*2/*m*) polymorph. The mol­ecular conformations of **1** and the two polymorphs of **2** are very similar, as all non-H atoms are planar except for the methyl of the ethyl groups. The inter­molecular inter­actions and crystal packing of **1** and **2** are described and compared with that of the reported monoclinic (*C*2/*m*) polymorph of derivative **2** [Castro Agudelo *et al.* (2017 ▸). *Acta Cryst.* E**73**, 1287–1289].

## Abstract 2

The synthesis, characterization and crystal structure of the title compounds are reported and the crystal supra­molecularity is discussed.

## Chemical context   

Cyano­acrylate derivatives are of industrial inter­est being subunits used to build many adhesives and polymeric materials (Faggi *et al.*, 2019 ▸). They are also considered important inter­mediate precursors for the synthesis of different heterocyclic derivatives, see for example Qian *et al.* (2018 ▸), and as nitrile-activated species in bioreduction reactions (Brenna *et al.*, 2013 ▸, 2015 ▸; Kong *et al.*, 2016 ▸) among others. In addition, they show important practical properties, such as in organic dye-sensitized solar cells (DSSCs) (He *et al.*, 2017 ▸; Zhou *et al.*, 2015 ▸). Within these voltaic cells, cyano­acrylic acid is one of the most commonly employed acceptors. Thio­phene and its deriv­atives, known to exhibit high charge mobility, serve as π-bridges (donor-π–acceptor structure) to provide conjugation and enhance light absorbance (Liu *et al.*, 2012 ▸).

An understanding of the structure of thio­phene-based acrylate subunits is necessary to benefit from their properties in photovoltaic cells. In a continuation of our work on the X-ray structural characterization of thio­phene-containing derivatives (Ibrahim *et al.*, 2019 ▸; Al-Refai *et al.*, 2014 ▸, 2016 ▸), we report here the synthesis, characterization and crystal structures of two thio­phene-based acrylate derivatives, namely, ethyl (*E*)-2-cyano-3-(3-methyl­thio­phen-2-yl)acrylate (**1**) and ethyl (*E*)-2-cyano-3-(3-methyl­thio­phen-2-yl)acrylate (**2**). Derivative **2** is a polymorph of a reported structure (Castro Agudelo *et al.*, 2017 ▸), but with no disorder of the eth­oxy group. The crystal supra­molecularity of both com­pounds is also discussed.
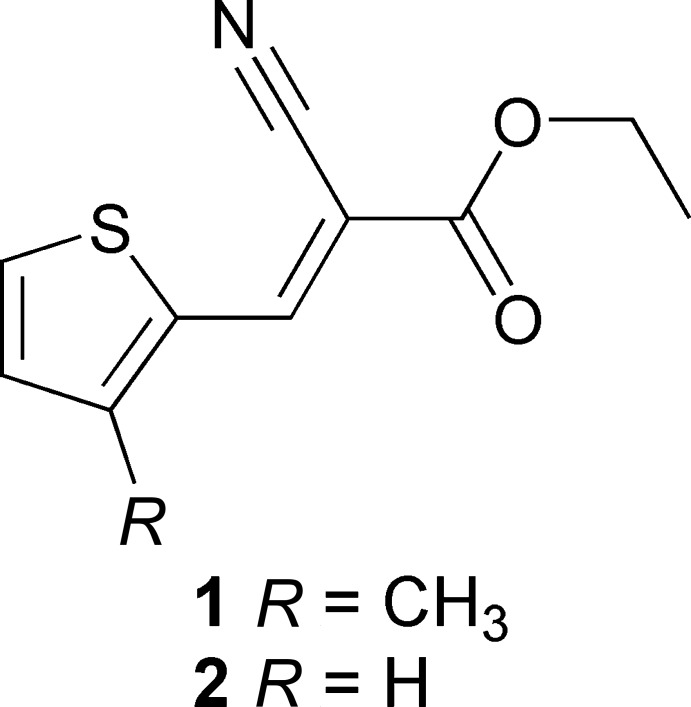


## Structural commentary   

**Figure 1:**
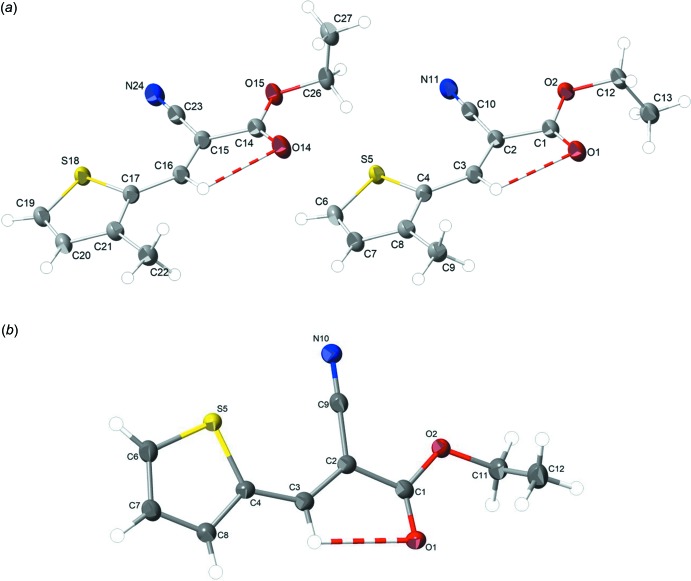
Mol­ecular structures of compounds (*a*) **1** and (*b*) **2** with the atom-labelling scheme (displacement ellipsoids at 50% probability level). Intra­molecular C—H⋯O inter­actions are presented as red–white multi-band cylinders.

The mol­ecular structures of the title compounds are depicted in Fig. 1 ▸. The asymmetric unit contains two independent mol­ecules, *A* and *B*, in **1** and one mol­ecule in **2**. In these mol­ecules, the bond distances and angles fall within similar ranges to those reported for similar compounds (Castro Agudelo *et al.*, 2017 ▸; Xu *et al.*, 2016 ▸). In both compounds, all non-hydrogen atoms, except for the methyl groups, lie nearly in the same planes. The differences in torsion angles [C1—C2—C3—C4 = −177.78 (14) and C1—O2—C12—C13 = 83.60 (15)° (mol­ecule *A*), C14—C15—C16—C17 = 179.71 (15)° and C14—O15—C26—C27 = −88.66 (2)° (mol­ecule *B*) in **1** and C1—C2—C3—C4 = −178.77 (11) and C1—O2—C11—C12 = −83.41 (13)° in **2**] indicate an out-of-plane deviation of the methyl group. The planarity of the mol­ecules allows intra­molecular hydrogen bonds to occur [C3—H3⋯O1 (mol­ecule *A*) and C16—H16⋯O14 (mol­ecule *B*) in **1**; C3—H3⋯O1 in **2**] (Fig. 1 ▸ and Tables 1 ▸ and 2 ▸), forming an *S*(6) ring motif with the carbonyl O and cyano N atoms consequently exhibiting an *anti*-configuration to each other. The conformation of the ethene bond is always *E* [C2=C3 = 1.363 (2) Å (mol­ecule *A*) and C15=C16 = 1.3625 (19) (mol­ecule *B*) in **1**; C2=C3 = 1.3592 (18) Å in **2**].

Derivative **2** is a polymorph of ethyl (*E*)-2-cyano-3-(­thio­phen-2-yl)acrylate (CSD refcode GEHYEA; Castro Agudelo *et al.*, 2017 ▸). It shows a similar structure to **1**, which has an extra methyl substituent on the thio­phene ring. In compound **1** and the two polymorphs of **2**, all thio­phene-based cyano­acrylate non-H atoms, except for the ethyl group, lie in the same plane. It is also noteworthy that in the polymorph, the ethyl fragment occurs in more than one conformation, thus resulting in disorder, which is absent in **1** and **2**.

## Supra­molecular features   

**Figure 2:**
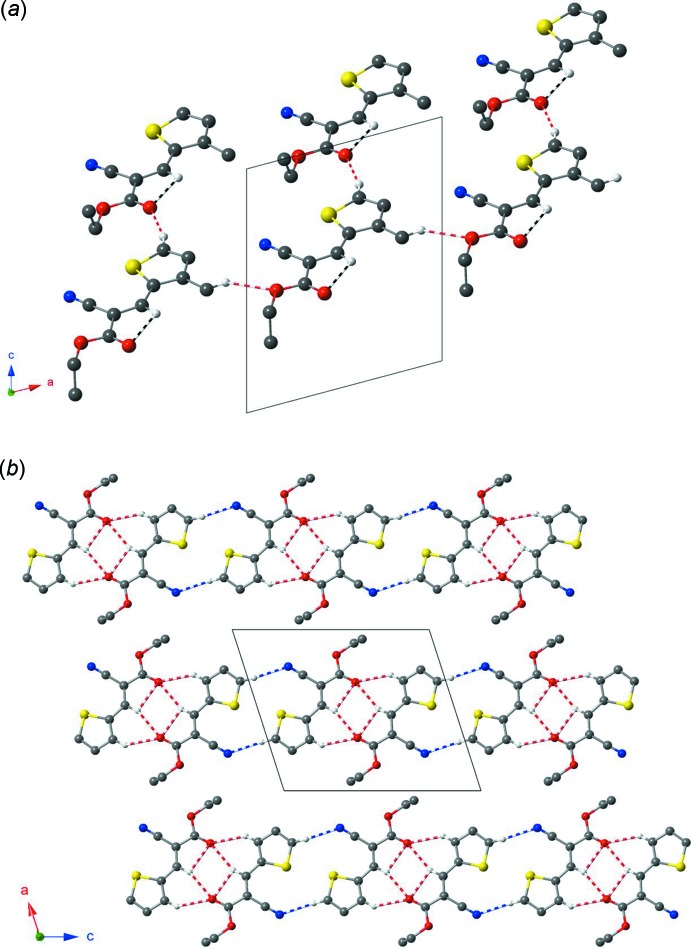
(*a*) Partial packing diagram for **1** showing layers of *A* and *B* mol­ecules parallel to the *ac* plane, and connected *via* C—H⋯O inter­molecular inter­actions (shown as multi-band cylinders). (*b*) The intra­molecular (black and white) and inter­molecular (red and white) inter­actions in **2** forming chains of dimeric species connected *via* C—H⋯O and C—H⋯N inter­actions. In both figures, hydrogen atoms not involved in inter­actions are omitted for clarity.

In the crystal of **1**, the *A* and *B* mol­ecules each form layers parallel to the *ac* plane, Fig. 2 ▸ *a*. The layers built up from chains of *B* mol­ecules are connected *via* C—H⋯O hydrogen bonds along the *a* axis. These chains are further connected through C—H⋯O inter­actions with stacks of mol­ecules *A* along the *c* axis. In the *b*-axis direction, inter­layered inter­actions through van der Waals forces and/or weak dipolar inter­actions generate a three-dimensional network. In the crystal of **2**, inversion dimers are assembled along the *c* axis through C—H⋯O inter­actions, Fig. 2 ▸ *b*. Adjacent dimers (along the *c* axis) are further connected through C—H⋯N inter­actions, leading to infinite chains propagating along the *c-*axis direction. The resulting chains interact via van der Waals forces to form sheets parallel to the *ac* plane (Fig. 2 ▸ *b*). The sheets are connected through van der Waals forces and/or weak dipolar inter­actions, thus consolidating the three-dimensional framework structure. Compounds **1**, **2** and the polymorph of **2** (Castro Agudelo *et al.*, 2017 ▸) show no apparent degree of π–π stacking.

The polymorph of **2** shows a similar crystal packing arrangement, the mol­ecules being connected *via* C—H⋯O/N inter­actions, generating centrosymmetric dimers. Chains of mol­ecules are further connected by van der Waals forces into sheets.

## Database survey   

Castro Agudelo *et al.* (2017 ▸) reported a recent survey on the Cambridge Structural Database [CSD Version 5.37 with two updates; Groom *et al.*, 2016 ▸] for hits containing the complete thio­phene-based cyano­acrylate fragment, together with the possibility of other five-membered rings and/or the presence of a saturated chain longer than the ethyl fragment. They found three hits containing the main part of the title compounds, the thio­phene-cyano­acrylate, with additional and/or longer substituents, namely ethyl-3-(3-chloro-4-cyano-5-{[4-(di­methyl­amino)­phen­yl]diazen­yl}-2-thien­yl)-2-cyano­acrylate (UMUYAE; Xu *et al.*, 2016 ▸), octyl-2-cyano-3-(4,6-di­bromo-7,7-dimethyl-7*H*-thieno[3′,4′:4,5]silolo[2,3-*b*]thio­phen-2-yl)acrylate (QUSKAS; Liu *et al.*, 2016 ▸) and ethyl-2-cyano-3-(3,3′′′-dihexyl-2,2′:5′,2′′:5′′,2′′′-quaterthio­phen-5-yl)acrylate (AVUFON; Miyazaki *et al.*, 2011 ▸). In all derivatives AVUFON, UMUYAE and QUSKAS, the non-H thio­phene-based acrylate fragment is almost planar except for the methyl group (or the longer alkyl chain in QUSKAS) being slightly out of the plane. The crystal lattices of AVUFON, UMUYAE and QUSKAS are stabilized by C—H⋯O/S, C—H⋯O/N and C—H⋯N/S inter­molecular inter­actions, respectively.

A further search of the CSD for other five-membered rings instead of thio­phene provided six hits. Of them, the following three are very similar to the title compounds: ethyl-(2*E*)-2-cyano-3-(1-methyl-1*H*-pyrrol-2-yl)prop-2-enoate (AYUGEH; Asiri *et al.*, 2011 ▸), (*E*)-ethyl-2-cyano-3-(1*H*-pyrrol-2-yl)acrylate (EVIZEP; Yuvaraj *et al.*, 2011 ▸) and (*E*)-ethyl-2-cyano-3-(furan-2-yl)acrylate (ZAQKIN; Kalkhambkar *et al.*, 2012 ▸). In both AYUGEH and EVIZEP, all the non-H atoms are nearly in the same plane, while in ZAQKIN the furan-based cyano­acrylate moiety lies in the same plane except for the methyl groups, which are slightly out of plane. As far as crystal packing is concerned, the mol­ecules in EVIZEP and ZAQKIN are linked into dimers *via* N—H⋯O and C—H⋯O hydrogen bonds, respectively, while in AYUGEH the mol­ecules are linked into tapes *via* both C—H⋯O and C—H⋯N inter­actions. The tapes are further inter­connected by C—H⋯*π* inter­actions into a three-dimensional structure.

## Synthesis and crystallization   

**Figure 3:**
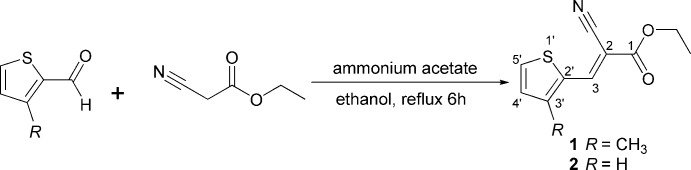
Syntheis of the title compounds.

All reagents and solvent were purchased from Aldrich and used without further purifications. The title compounds were synthesized as outlined in Fig. 3 ▸.

In a 250 ml round-bottom flask connected with a condenser, a mixture of the corresponding thio­phene-2-carboxaldehyde (1 mmol), ethyl­cyano­acetate (1.1 mmol) and ammonium acetate (8 mmol) in absolute ethanol was refluxed for 6 h. The reaction was monitored using thin layer chromatography (TLC plates coated with silica gel). After completion, the reaction mixture was cooled to room temperature, and the obtained yellowish-brown precipitate was filtered off, washed with cooled water, dried and recrystallized from ethanol solution to give the final products as pale-yellow crystals (90% yield for both **1** and **2**).

Ethyl (*E*)-2-cyano-3-(3-methyl­thio­phen-2-yl)acrylate (**1**): m.p. 381–382 K, ^1^H NMR (CD_2_Cl_2_, 300 MHz): δ (ppm) = 1.39 (*t*, *J* = 7.12, 3H, CH_2_CH_3_), 2.48 (*s*, 3H, CH_3_-3′), 4.36 (*q*, *J* = 7.12, 2H, CH_2_CH_3_), 7.07 (*d*, *J* = 5.01,1H, H-4′), 7.74 (*d*, *J* = 5.01, 1H, H-5′), 8.46 (*s*, 1H, H-3). ^13^C NMR (CD_2_Cl_2_, 75 MHz) δ (ppm) = 14.4 (CH_2_CH_3_), 14.9 (CH_3_-3′), 62.7 (CH_2_CH_3_), 98.0 (C-2), 116.4 (CN), 131.2 (C-2′), 131.4 (C-4′), 134.3 (C-5′), 145.0 (C-3), 149.9 (C-3′), 163.4 (C-1). (+)-ESIMS *m*/*z* = 244 ([*M* + Na]^+^, 100%), 465 ([2*M* + Na]^+^, 16%).

Ethyl (*E*)-2-cyano-3-(thio­phen-2-yl)acrylate (**2**): mp. 371–372 K, ^1^H NMR (CD_2_Cl_2_, 300 MHz): δ (ppm) = 1.39 (*t*, *J* = 7.12, 3H, CH_2_CH_3_), 4.37 (*q*, *J* = 7.12, 2H, CH_2_CH_3_), 7.27 (*t*, *J* = 4.44,1H, H-4′), 7.85 (*d*, *J* = 4.36, 2H, H-3′,5′), 8.38 (*s*, 1H, H-3). ^13^C NMR (CD_2_Cl_2_, 75 MHz) δ (ppm) = 14.4 (CH_2_CH_3_), 62.9 (CH_2_CH_3_), 99.8 (C-2), 116.1 (CN), 129.0 (C-4′), 135.5 (C-5′), 136.5 (C-2′), 137.8 (C-3′), 146.9 (C-3), 162.9 (C-1). (+)-ESIMS *m*/*z* = 230 ([*M* + Na]^+^, 100%), 237 ([2*M* + Na]^+^, 11%).

## Refinement   

Detailed crystal data and structure refinement for the title compounds are listed in Table 3 ▸. In **1**, C-bound hydrogen atoms were included in calculated positions (0.95–0.99 Å) and refined using a riding model with *U*_iso_(H) = 1.2*U*_eq_(C) or 1.5*U*_eq_(C-meth­yl). Methyl groups were allowed to rotate to fit best the electron density. All hydrogen atoms in **2** were located in difference-Fourier maps and refined isotropically.

## Supplementary Material

Crystal structure: contains datablock(s) 1, 2, New_Global_Publ_Block. DOI: 10.1107/S2056989019011435/tx2012sup1.cif

Structure factors: contains datablock(s) 1. DOI: 10.1107/S2056989019011435/tx20121sup2.hkl

Structure factors: contains datablock(s) 2. DOI: 10.1107/S2056989019011435/tx20122sup3.hkl

Supporting information file. DOI: 10.1107/S2056989019011435/tx20121sup4.cml

Supporting information file. DOI: 10.1107/S2056989019011435/tx20122sup5.cml

Supporting information file. DOI: 10.1107/S2056989019011435/tx2012sup6.pdf

CCDC references: 1947083, 1947082

Additional supporting information:  crystallographic information; 3D view; checkCIF report

## Figures and Tables

**Table 1:** Hydrogen-bond geometry (Å, °) for **1** [Chem scheme1]

*D*—H⋯*A*	*D*—H	H⋯*A*	*D*⋯*A*	*D*—H⋯*A*
C3—H3⋯O1	0.95	2.43	2.8136 (19)	104
C16—H16⋯O14	0.95	2.38	2.7829 (19)	105
C19—H19⋯O1^i^	0.95	2.34	3.2708 (19)	165
C22—H22*A*⋯O15^ii^	0.98	2.59	3.292 (2)	128

Symmetry codes: (i) 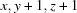; (ii) 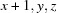.

**Table 2:** Hydrogen-bond geometry (Å, °) for **2** [Chem scheme1]

*D*—H⋯*A*	*D*—H	H⋯*A*	*D*⋯*A*	*D*—H⋯*A*
C3—H3⋯O1	0.964 (19)	2.444 (19)	2.7998 (18)	101.5 (14)
C3—H3⋯O1^i^	0.964 (19)	2.45 (2)	3.3436 (18)	153.4 (15)
C6—H6⋯N10^ii^	0.99 (2)	2.49 (2)	3.465 (2)	169.1 (18)
C8—H8⋯O1^i^	0.94 (2)	2.50 (2)	3.3047 (19)	143.6 (17)

Symmetry codes: (i) 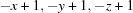; (ii) 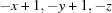.

**Table 3:** Experimental details

	**1**	**2**
Crystal data		
Chemical formula	C_11_H_11_NO_2_S	C_10_H_9_NO_2_S
*M*_r_	221.27	207.24
Crystal system, space group	Triclinic, *P* 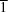	Monoclinic, *P*2_1_/*c*
Temperature (K)	100	100
*a*, *b*, *c* (Å)	9.2784 (2), 10.7925 (3), 11.6696 (2)	11.5907 (3), 6.6883 (2), 13.4837 (3)
α, β, γ (°)	74.464 (2), 74.179 (2), 85.073 (2)	90, 107.859 (2), 90
*V* (Å^3^)	1083.11 (4)	994.92 (5)
*Z*	4	4
Radiation type	Cu *K*α	Cu *K*α
μ (mm^−1^)	2.49	2.68
Crystal size (mm)	0.26 × 0.24 × 0.11	0.32 × 0.20 × 0.20

Data collection		
Diffractometer	Stoe STADIVARI	Stoe STADIVARI
Absorption correction	Multi-scan (*LANA*; Stoe & Cie, 2016 ▸)	Multi-scan (*LANA*; Stoe & Cie, 2016 ▸)
*T*_min_, *T*_max_	0.074, 0.546	0.051, 0.168
No. of measured, independent and observed [*I* > 2σ(*I*)] reflections	21624, 4399, 3911	10400, 2048, 1974
*R*_int_	0.032	0.026
(sin θ/λ)_max_ (Å^−1^)	0.630	0.630

Refinement		
*R*[*F* ^2^ > 2σ(*F* ^2^)], *wR*(*F* ^2^), *S*	0.041, 0.122, 1.10	0.033, 0.100, 1.09
No. of reflections	4399	2048
No. of parameters	275	164
H-atom treatment	H-atom parameters constrained	All H-atom parameters refined
Δρ_max_, Δρ_min_ (e Å^−3^)	0.37, −0.49	0.29, −0.28

Computer programs: *X-AREA Pilatus*, *Recipe* and *Integrate* (Stoe & Cie, 2016 ▸) *SHELXT2014* (Sheldrick, 2015*a* ▸), *SHELXL2018* (Sheldrick, 2015*b* ▸) and *DIAMOND* (Crystal Impact, 2014 ▸).

## supplementary crystallographic information

### Ethyl (E)-2-cyano-3-(3-methylthiophen-2-yl)acrylate (1) Crystal data

**Table:**

C_11_H_11_NO_2_S	*Z* = 4
*M**_r_* = 221.27	*F*(000) = 464
Triclinic, *P*1	*D*_x_ = 1.357 Mg m^−^^3^
*a* = 9.2784 (2) Å	Cu *K*α radiation, λ = 1.54186 Å
*b* = 10.7925 (3) Å	Cell parameters from 24144 reflections
*c* = 11.6696 (2) Å	θ = 4.3–76.6°
α = 74.464 (2)°	µ = 2.49 mm^−^^1^
β = 74.179 (2)°	*T* = 100 K
γ = 85.073 (2)°	Plate, colourless
*V* = 1083.11 (4) Å^3^	0.26 × 0.24 × 0.11 mm

### Ethyl (E)-2-cyano-3-(3-methylthiophen-2-yl)acrylate (1) Data collection

**Table:**

Stoe STADIVARI diffractometer	4399 independent reflections
Radiation source: GeniX 3D HF Cu	3911 reflections with *I* > 2σ(*I*)
Detector resolution: 5.81 pixels mm^-1^	*R*_int_ = 0.032
rotation method, ω scans	θ_max_ = 76.1°, θ_min_ = 4.3°
Absorption correction: multi-scan (*LANA*; Stoe & Cie, 2016)	*h* = −11→11
*T*_min_ = 0.074, *T*_max_ = 0.546	*k* = −9→13
21624 measured reflections	*l* = −14→14

### Ethyl (E)-2-cyano-3-(3-methylthiophen-2-yl)acrylate (1) Refinement

**Table:**

Refinement on *F*^2^	Primary atom site location: dual
Least-squares matrix: full	Secondary atom site location: difference Fourier map
*R*[*F*^2^ > 2σ(*F*^2^)] = 0.041	Hydrogen site location: inferred from neighbouring sites
*wR*(*F*^2^) = 0.122	H-atom parameters constrained
*S* = 1.10	*w* = 1/[σ^2^(*F*_o_^2^) + (0.0848*P*)^2^ + 0.1177*P*] where *P* = (*F*_o_^2^ + 2*F*_c_^2^)/3
4399 reflections	(Δ/σ)_max_ = 0.001
275 parameters	Δρ_max_ = 0.37 e Å^−^^3^
0 restraints	Δρ_min_ = −0.49 e Å^−^^3^

### Ethyl (E)-2-cyano-3-(3-methylthiophen-2-yl)acrylate (1) Special details

**Table:**

Geometry. All esds (except the esd in the dihedral angle between two l.s. planes) are estimated using the full covariance matrix. The cell esds are taken into account individually in the estimation of esds in distances, angles and torsion angles; correlations between esds in cell parameters are only used when they are defined by crystal symmetry. An approximate (isotropic) treatment of cell esds is used for estimating esds involving l.s. planes.

### Ethyl (E)-2-cyano-3-(3-methylthiophen-2-yl)acrylate (1) Fractional atomic coordinates and isotropic or equivalent isotropic displacement parameters (Å^2^)

**Table:**

	*x*	*y*	*z*	*U*_iso_*/*U*_eq_	
O1	0.50743 (12)	0.18612 (11)	−0.05309 (9)	0.0282 (2)	
C1	0.41104 (16)	0.24976 (14)	−0.00171 (13)	0.0244 (3)	
O2	0.26787 (11)	0.25753 (11)	−0.00683 (9)	0.0272 (2)	
C2	0.43611 (16)	0.33079 (14)	0.07627 (13)	0.0241 (3)	
C3	0.57815 (16)	0.33732 (14)	0.08609 (13)	0.0237 (3)	
H3	0.651544	0.290986	0.038820	0.028*	
C4	0.63437 (16)	0.40276 (14)	0.15613 (13)	0.0237 (3)	
S5	0.52472 (4)	0.48985 (3)	0.25473 (3)	0.02482 (13)	
C6	0.67767 (17)	0.52373 (15)	0.29500 (13)	0.0279 (3)	
H6	0.673143	0.573149	0.352151	0.033*	
C7	0.80743 (16)	0.47210 (15)	0.23616 (13)	0.0271 (3)	
H7	0.902616	0.481512	0.248382	0.033*	
C8	0.78481 (16)	0.40304 (14)	0.15496 (13)	0.0253 (3)	
C9	0.90876 (16)	0.33949 (15)	0.07698 (14)	0.0288 (3)	
H9A	1.002445	0.344255	0.098973	0.043*	
H9B	0.920518	0.383536	−0.010108	0.043*	
H9C	0.884318	0.249167	0.091128	0.043*	
C10	0.30968 (16)	0.39583 (15)	0.13711 (13)	0.0260 (3)	
N11	0.20792 (14)	0.44850 (13)	0.18585 (12)	0.0307 (3)	
C12	0.22775 (17)	0.18030 (16)	−0.07799 (14)	0.0293 (3)	
H12A	0.311397	0.179597	−0.151655	0.035*	
H12B	0.138713	0.218888	−0.106211	0.035*	
C13	0.19407 (17)	0.04420 (16)	−0.00130 (15)	0.0335 (3)	
H13A	0.154960	−0.003295	−0.046871	0.050*	
H13B	0.119282	0.045515	0.076064	0.050*	
H13C	0.286108	0.002013	0.016692	0.050*	
O14	0.39990 (12)	0.67602 (12)	0.40403 (10)	0.0326 (3)	
C14	0.29946 (16)	0.72830 (15)	0.46523 (13)	0.0262 (3)	
O15	0.15438 (11)	0.72446 (11)	0.46857 (10)	0.0285 (2)	
C15	0.32273 (16)	0.80391 (14)	0.54804 (13)	0.0251 (3)	
C16	0.46568 (16)	0.81134 (14)	0.55532 (12)	0.0246 (3)	
H16	0.538142	0.766223	0.506059	0.029*	
C17	0.52508 (16)	0.87543 (14)	0.62444 (13)	0.0245 (3)	
S18	0.42127 (4)	0.96642 (3)	0.72124 (3)	0.02552 (13)	
C19	0.57711 (17)	0.99953 (15)	0.75821 (13)	0.0282 (3)	
H19	0.575810	1.050830	0.813150	0.034*	
C20	0.70388 (17)	0.94398 (15)	0.69991 (13)	0.0279 (3)	
H20	0.800387	0.952569	0.710077	0.033*	
C22	0.79644 (17)	0.80089 (16)	0.54916 (14)	0.0306 (3)	
H22A	0.894192	0.818438	0.558037	0.046*	
H22B	0.796091	0.829397	0.462132	0.046*	
H22C	0.777504	0.708354	0.579387	0.046*	
C21	0.67641 (16)	0.87201 (15)	0.62249 (13)	0.0258 (3)	
C23	0.19658 (16)	0.86371 (15)	0.61513 (13)	0.0271 (3)	
N24	0.09612 (15)	0.91263 (14)	0.67014 (12)	0.0324 (3)	
C26	0.11875 (17)	0.65600 (15)	0.38777 (14)	0.0300 (3)	
H26A	0.017243	0.619428	0.424811	0.036*	
H26B	0.191159	0.584110	0.378726	0.036*	
C27	0.12498 (19)	0.74571 (17)	0.26288 (14)	0.0360 (4)	
H27A	0.092643	0.700287	0.212052	0.054*	
H27B	0.227878	0.775245	0.222919	0.054*	
H27C	0.058482	0.819932	0.272496	0.054*	

### Ethyl (E)-2-cyano-3-(3-methylthiophen-2-yl)acrylate (1) Atomic displacement parameters (Å^2^)

**Table:**

	*U*^11^	*U*^22^	*U*^33^	*U*^12^	*U*^13^	*U*^23^
O1	0.0258 (5)	0.0336 (6)	0.0305 (5)	0.0029 (4)	−0.0085 (4)	−0.0166 (4)
C1	0.0237 (7)	0.0275 (8)	0.0244 (6)	0.0005 (5)	−0.0079 (5)	−0.0096 (6)
O2	0.0232 (5)	0.0346 (6)	0.0320 (5)	0.0020 (4)	−0.0115 (4)	−0.0185 (4)
C2	0.0237 (7)	0.0268 (8)	0.0249 (6)	0.0014 (5)	−0.0086 (5)	−0.0101 (6)
C3	0.0238 (7)	0.0258 (7)	0.0237 (6)	0.0005 (5)	−0.0065 (5)	−0.0098 (5)
C4	0.0230 (7)	0.0265 (8)	0.0253 (6)	0.0019 (5)	−0.0076 (5)	−0.0121 (5)
S5	0.0228 (2)	0.0302 (2)	0.0267 (2)	0.00155 (14)	−0.00801 (14)	−0.01489 (15)
C6	0.0293 (7)	0.0315 (8)	0.0290 (7)	−0.0017 (6)	−0.0115 (6)	−0.0135 (6)
C7	0.0253 (7)	0.0313 (8)	0.0291 (7)	−0.0012 (6)	−0.0101 (6)	−0.0115 (6)
C8	0.0244 (7)	0.0282 (8)	0.0251 (6)	0.0002 (5)	−0.0071 (5)	−0.0095 (6)
C9	0.0226 (7)	0.0357 (9)	0.0311 (7)	0.0029 (6)	−0.0068 (6)	−0.0149 (6)
C10	0.0256 (7)	0.0302 (8)	0.0280 (6)	0.0005 (6)	−0.0117 (5)	−0.0126 (6)
N11	0.0263 (6)	0.0384 (8)	0.0342 (6)	0.0030 (5)	−0.0103 (5)	−0.0190 (6)
C12	0.0282 (7)	0.0361 (9)	0.0325 (7)	0.0023 (6)	−0.0134 (6)	−0.0188 (6)
C13	0.0303 (7)	0.0382 (9)	0.0393 (8)	−0.0019 (6)	−0.0111 (6)	−0.0194 (7)
O14	0.0289 (5)	0.0416 (7)	0.0359 (6)	0.0049 (5)	−0.0102 (4)	−0.0239 (5)
C14	0.0258 (7)	0.0296 (8)	0.0264 (7)	0.0007 (6)	−0.0088 (5)	−0.0105 (6)
O15	0.0249 (5)	0.0360 (6)	0.0312 (5)	−0.0003 (4)	−0.0092 (4)	−0.0175 (4)
C15	0.0252 (7)	0.0285 (8)	0.0248 (6)	0.0017 (5)	−0.0076 (5)	−0.0116 (5)
C16	0.0255 (7)	0.0277 (8)	0.0232 (6)	0.0005 (5)	−0.0068 (5)	−0.0106 (5)
C17	0.0247 (7)	0.0278 (8)	0.0242 (6)	0.0014 (5)	−0.0068 (5)	−0.0121 (5)
S18	0.0248 (2)	0.0310 (2)	0.0255 (2)	0.00185 (14)	−0.00731 (14)	−0.01494 (15)
C19	0.0319 (7)	0.0306 (8)	0.0273 (7)	−0.0015 (6)	−0.0111 (6)	−0.0124 (6)
C20	0.0281 (7)	0.0322 (8)	0.0283 (7)	−0.0023 (6)	−0.0108 (6)	−0.0119 (6)
C22	0.0261 (7)	0.0364 (9)	0.0325 (7)	0.0043 (6)	−0.0077 (6)	−0.0158 (6)
C21	0.0245 (7)	0.0294 (8)	0.0259 (7)	0.0005 (6)	−0.0073 (5)	−0.0105 (6)
C23	0.0268 (7)	0.0312 (8)	0.0278 (7)	0.0002 (6)	−0.0105 (6)	−0.0117 (6)
N24	0.0272 (6)	0.0403 (8)	0.0342 (7)	0.0025 (5)	−0.0078 (5)	−0.0178 (6)
C26	0.0300 (7)	0.0338 (9)	0.0337 (8)	−0.0012 (6)	−0.0112 (6)	−0.0179 (6)
C27	0.0377 (8)	0.0441 (10)	0.0341 (8)	−0.0029 (7)	−0.0139 (6)	−0.0179 (7)

### Ethyl (E)-2-cyano-3-(3-methylthiophen-2-yl)acrylate (1) Geometric parameters (Å, º)

**Table:**

O1—C1	1.2057 (17)	O14—C14	1.2081 (18)
C1—O2	1.3404 (17)	C14—O15	1.3399 (17)
C1—C2	1.4912 (19)	C14—C15	1.4876 (19)
O2—C12	1.4545 (16)	O15—C26	1.4593 (16)
C2—C3	1.363 (2)	C15—C16	1.3625 (19)
C2—C10	1.4298 (19)	C15—C23	1.428 (2)
C3—C4	1.4296 (19)	C16—C17	1.4282 (19)
C3—H3	0.9500	C16—H16	0.9500
C4—C8	1.3925 (19)	C17—C21	1.3955 (19)
C4—S5	1.7375 (14)	C17—S18	1.7339 (14)
S5—C6	1.7068 (14)	S18—C19	1.7076 (15)
C6—C7	1.368 (2)	C19—C20	1.366 (2)
C6—H6	0.9500	C19—H19	0.9500
C7—C8	1.4181 (19)	C20—C21	1.421 (2)
C7—H7	0.9500	C20—H20	0.9500
C8—C9	1.4997 (19)	C22—C21	1.499 (2)
C9—H9A	0.9800	C22—H22A	0.9800
C9—H9B	0.9800	C22—H22B	0.9800
C9—H9C	0.9800	C22—H22C	0.9800
C10—N11	1.1517 (19)	C23—N24	1.153 (2)
C12—C13	1.509 (2)	C26—C27	1.508 (2)
C12—H12A	0.9900	C26—H26A	0.9900
C12—H12B	0.9900	C26—H26B	0.9900
C13—H13A	0.9800	C27—H27A	0.9800
C13—H13B	0.9800	C27—H27B	0.9800
C13—H13C	0.9800	C27—H27C	0.9800
			
O1—C1—O2	124.91 (13)	O14—C14—O15	124.76 (13)
O1—C1—C2	124.13 (13)	O14—C14—C15	123.55 (13)
O2—C1—C2	110.96 (12)	O15—C14—C15	111.68 (12)
C1—O2—C12	116.44 (11)	C14—O15—C26	116.44 (11)
C3—C2—C10	123.99 (13)	C16—C15—C23	123.75 (13)
C3—C2—C1	117.90 (13)	C16—C15—C14	117.06 (13)
C10—C2—C1	118.10 (12)	C23—C15—C14	119.19 (12)
C2—C3—C4	130.33 (14)	C15—C16—C17	130.94 (14)
C2—C3—H3	114.8	C15—C16—H16	114.5
C4—C3—H3	114.8	C17—C16—H16	114.5
C8—C4—C3	123.93 (13)	C21—C17—C16	123.66 (14)
C8—C4—S5	111.36 (10)	C21—C17—S18	111.12 (11)
C3—C4—S5	124.71 (11)	C16—C17—S18	125.22 (11)
C6—S5—C4	91.34 (7)	C19—S18—C17	91.77 (7)
C7—C6—S5	112.77 (11)	C20—C19—S18	112.44 (11)
C7—C6—H6	123.6	C20—C19—H19	123.8
S5—C6—H6	123.6	S18—C19—H19	123.8
C6—C7—C8	112.84 (13)	C19—C20—C21	113.02 (13)
C6—C7—H7	123.6	C19—C20—H20	123.5
C8—C7—H7	123.6	C21—C20—H20	123.5
C4—C8—C7	111.68 (13)	C21—C22—H22A	109.5
C4—C8—C9	124.57 (13)	C21—C22—H22B	109.5
C7—C8—C9	123.75 (13)	H22A—C22—H22B	109.5
C8—C9—H9A	109.5	C21—C22—H22C	109.5
C8—C9—H9B	109.5	H22A—C22—H22C	109.5
H9A—C9—H9B	109.5	H22B—C22—H22C	109.5
C8—C9—H9C	109.5	C17—C21—C20	111.66 (13)
H9A—C9—H9C	109.5	C17—C21—C22	124.81 (13)
H9B—C9—H9C	109.5	C20—C21—C22	123.53 (13)
N11—C10—C2	179.8 (2)	N24—C23—C15	178.96 (15)
O2—C12—C13	110.63 (12)	O15—C26—C27	110.46 (12)
O2—C12—H12A	109.5	O15—C26—H26A	109.6
C13—C12—H12A	109.5	C27—C26—H26A	109.6
O2—C12—H12B	109.5	O15—C26—H26B	109.6
C13—C12—H12B	109.5	C27—C26—H26B	109.6
H12A—C12—H12B	108.1	H26A—C26—H26B	108.1
C12—C13—H13A	109.5	C26—C27—H27A	109.5
C12—C13—H13B	109.5	C26—C27—H27B	109.5
H13A—C13—H13B	109.5	H27A—C27—H27B	109.5
C12—C13—H13C	109.5	C26—C27—H27C	109.5
H13A—C13—H13C	109.5	H27A—C27—H27C	109.5
H13B—C13—H13C	109.5	H27B—C27—H27C	109.5
			
O1—C1—O2—C12	1.4 (2)	O14—C14—O15—C26	−2.6 (2)
C2—C1—O2—C12	−178.63 (12)	C15—C14—O15—C26	178.16 (12)
O1—C1—C2—C3	2.7 (2)	O14—C14—C15—C16	−1.2 (2)
O2—C1—C2—C3	−177.20 (13)	O15—C14—C15—C16	178.04 (13)
O1—C1—C2—C10	−176.67 (14)	O14—C14—C15—C23	179.03 (15)
O2—C1—C2—C10	3.39 (18)	O15—C14—C15—C23	−1.8 (2)
C10—C2—C3—C4	1.6 (3)	C23—C15—C16—C17	−0.5 (3)
C1—C2—C3—C4	−177.78 (14)	C14—C15—C16—C17	179.71 (15)
C2—C3—C4—C8	−178.52 (15)	C15—C16—C17—C21	179.19 (16)
C2—C3—C4—S5	2.4 (2)	C15—C16—C17—S18	−0.6 (2)
C8—C4—S5—C6	−0.81 (12)	C21—C17—S18—C19	0.10 (12)
C3—C4—S5—C6	178.37 (14)	C16—C17—S18—C19	179.92 (14)
C4—S5—C6—C7	0.29 (12)	C17—S18—C19—C20	−0.07 (13)
S5—C6—C7—C8	0.30 (17)	S18—C19—C20—C21	0.02 (18)
C3—C4—C8—C7	−178.06 (14)	C16—C17—C21—C20	−179.93 (14)
S5—C4—C8—C7	1.12 (16)	S18—C17—C21—C20	−0.11 (17)
C3—C4—C8—C9	2.5 (2)	C16—C17—C21—C22	−0.4 (2)
S5—C4—C8—C9	−178.36 (12)	S18—C17—C21—C22	179.43 (12)
C6—C7—C8—C4	−0.93 (19)	C19—C20—C21—C17	0.1 (2)
C6—C7—C8—C9	178.56 (14)	C19—C20—C21—C22	−179.49 (14)
C1—O2—C12—C13	83.60 (15)	C14—O15—C26—C27	−88.66 (16)

### Ethyl (E)-2-cyano-3-(3-methylthiophen-2-yl)acrylate (1) Hydrogen-bond geometry (Å, º)

**Table:**

*D*—H···*A*	*D*—H	H···*A*	*D*···*A*	*D*—H···*A*
C3—H3···O1	0.95	2.43	2.8136 (19)	104
C16—H16···O14	0.95	2.38	2.7829 (19)	105
C19—H19···O1^i^	0.95	2.34	3.2708 (19)	165
C22—H22*A*···O15^ii^	0.98	2.59	3.292 (2)	128

Symmetry codes: (i) *x*, *y*+1, *z*+1; (ii) *x*+1, *y*, *z*.

### Ethyl (E)-2-cyano-3-(thiophen-2-yl)acrylate (2) Crystal data

**Table:**

C_10_H_9_NO_2_S	*F*(000) = 432
*M**_r_* = 207.24	*D*_x_ = 1.384 Mg m^−^^3^
Monoclinic, *P*2_1_/*c*	Cu *K*α radiation, λ = 1.54186 Å
*a* = 11.5907 (3) Å	Cell parameters from 15380 reflections
*b* = 6.6883 (2) Å	θ = 3.5–76.4°
*c* = 13.4837 (3) Å	µ = 2.68 mm^−^^1^
β = 107.859 (2)°	*T* = 100 K
*V* = 994.92 (5) Å^3^	Block, colourless
*Z* = 4	0.32 × 0.20 × 0.20 mm

### Ethyl (E)-2-cyano-3-(thiophen-2-yl)acrylate (2) Data collection

**Table:**

Stoe STADIVARI diffractometer	2048 independent reflections
Radiation source: GeniX 3D HF Cu	1974 reflections with *I* > 2σ(*I*)
Detector resolution: 5.81 pixels mm^-1^	*R*_int_ = 0.026
rotation method, ω scans	θ_max_ = 76.3°, θ_min_ = 6.8°
Absorption correction: multi-scan (*LANA*; Stoe & Cie, 2016)	*h* = −14→14
*T*_min_ = 0.051, *T*_max_ = 0.168	*k* = −8→8
10400 measured reflections	*l* = −16→10

### Ethyl (E)-2-cyano-3-(thiophen-2-yl)acrylate (2) Refinement

**Table:**

Refinement on *F*^2^	Secondary atom site location: difference Fourier map
Least-squares matrix: full	Hydrogen site location: difference Fourier map
*R*[*F*^2^ > 2σ(*F*^2^)] = 0.033	All H-atom parameters refined
*wR*(*F*^2^) = 0.100	*w* = 1/[σ^2^(*F*_o_^2^) + (0.0688*P*)^2^ + 0.2209*P*] where *P* = (*F*_o_^2^ + 2*F*_c_^2^)/3
*S* = 1.09	(Δ/σ)_max_ < 0.001
2048 reflections	Δρ_max_ = 0.29 e Å^−^^3^
164 parameters	Δρ_min_ = −0.28 e Å^−^^3^
0 restraints	Extinction correction: SHELXL-2018/3 (Sheldrick 2015), Fc^*^=kFc[1+0.001xFc^2^λ^3^/sin(2θ)]^-1/4^
Primary atom site location: dual	Extinction coefficient: 0.0030 (6)

### Ethyl (E)-2-cyano-3-(thiophen-2-yl)acrylate (2) Special details

**Table:**

Geometry. All esds (except the esd in the dihedral angle between two l.s. planes) are estimated using the full covariance matrix. The cell esds are taken into account individually in the estimation of esds in distances, angles and torsion angles; correlations between esds in cell parameters are only used when they are defined by crystal symmetry. An approximate (isotropic) treatment of cell esds is used for estimating esds involving l.s. planes.

### Ethyl (E)-2-cyano-3-(thiophen-2-yl)acrylate (2) Fractional atomic coordinates and isotropic or equivalent isotropic displacement parameters (Å^2^)

**Table:**

	*x*	*y*	*z*	*U*_iso_*/*U*_eq_	
O1	0.67411 (9)	0.47068 (14)	0.54042 (8)	0.0266 (3)	
C1	0.72087 (12)	0.46539 (17)	0.47233 (11)	0.0214 (3)	
O2	0.84079 (8)	0.45263 (13)	0.48771 (8)	0.0238 (2)	
C2	0.65241 (12)	0.46897 (17)	0.35943 (11)	0.0211 (3)	
C3	0.52935 (12)	0.47114 (18)	0.33171 (11)	0.0215 (3)	
H3	0.4938 (17)	0.475 (2)	0.3876 (14)	0.025 (4)*	
C4	0.44161 (12)	0.47115 (17)	0.23001 (11)	0.0210 (3)	
S5	0.47197 (3)	0.46815 (5)	0.11198 (2)	0.02218 (16)	
C6	0.32131 (13)	0.47018 (18)	0.04368 (12)	0.0250 (3)	
H6	0.301 (2)	0.470 (3)	−0.0329 (19)	0.044 (6)*	
C7	0.24983 (13)	0.47201 (19)	0.10750 (12)	0.0256 (3)	
H7	0.158 (2)	0.472 (3)	0.0817 (19)	0.053 (6)*	
C8	0.31729 (13)	0.47269 (18)	0.21394 (12)	0.0229 (3)	
H8	0.2819 (18)	0.476 (2)	0.2678 (16)	0.031 (5)*	
C9	0.71814 (12)	0.46836 (18)	0.28517 (11)	0.0223 (3)	
H9	0.8744 (14)	0.354 (3)	0.6320 (12)	0.026 (4)*	
N10	0.76940 (11)	0.46866 (17)	0.22440 (10)	0.0272 (3)	
H10	0.9901 (15)	0.379 (3)	0.5923 (13)	0.029 (4)*	
C11	0.91574 (13)	0.4415 (2)	0.59654 (12)	0.0277 (3)	
H11	0.8636 (16)	0.712 (3)	0.6428 (13)	0.039 (5)*	
C12	0.94012 (13)	0.6466 (3)	0.64456 (12)	0.0355 (3)	
H12	0.9946 (17)	0.630 (3)	0.7176 (16)	0.051 (5)*	
H13	0.9802 (15)	0.731 (3)	0.6063 (14)	0.044 (5)*	

### Ethyl (E)-2-cyano-3-(thiophen-2-yl)acrylate (2) Atomic displacement parameters (Å^2^)

**Table:**

	*U*^11^	*U*^22^	*U*^33^	*U*^12^	*U*^13^	*U*^23^
O1	0.0208 (5)	0.0392 (6)	0.0210 (5)	0.0009 (4)	0.0081 (4)	0.0005 (4)
C1	0.0179 (6)	0.0224 (6)	0.0235 (7)	−0.0005 (4)	0.0055 (5)	−0.0001 (4)
O2	0.0170 (5)	0.0334 (5)	0.0205 (5)	0.0013 (3)	0.0050 (4)	0.0002 (3)
C2	0.0196 (6)	0.0236 (6)	0.0203 (7)	0.0004 (4)	0.0062 (5)	0.0002 (4)
C3	0.0205 (7)	0.0220 (7)	0.0225 (7)	0.0002 (4)	0.0071 (5)	−0.0001 (4)
C4	0.0202 (7)	0.0236 (6)	0.0203 (7)	−0.0006 (4)	0.0077 (5)	−0.0004 (4)
S5	0.0193 (2)	0.0285 (2)	0.0190 (2)	−0.00064 (10)	0.00624 (14)	−0.00010 (10)
C6	0.0221 (6)	0.0269 (7)	0.0234 (7)	−0.0009 (5)	0.0033 (5)	0.0002 (5)
C7	0.0196 (6)	0.0290 (7)	0.0264 (8)	−0.0006 (5)	0.0046 (5)	0.0004 (5)
C8	0.0210 (7)	0.0240 (6)	0.0236 (7)	−0.0003 (4)	0.0068 (5)	−0.0001 (4)
C9	0.0178 (6)	0.0245 (7)	0.0231 (7)	0.0000 (4)	0.0040 (5)	0.0003 (4)
N10	0.0213 (6)	0.0371 (7)	0.0236 (6)	0.0006 (4)	0.0073 (5)	0.0007 (4)
C11	0.0195 (6)	0.0398 (7)	0.0212 (7)	0.0042 (5)	0.0024 (5)	0.0014 (5)
C12	0.0229 (6)	0.0484 (9)	0.0318 (8)	0.0018 (6)	0.0032 (5)	−0.0091 (7)

### Ethyl (E)-2-cyano-3-(thiophen-2-yl)acrylate (2) Geometric parameters (Å, º)

**Table:**

O1—C1	1.2012 (18)	C6—H6	0.99 (2)
C1—O2	1.3438 (15)	C7—C8	1.408 (2)
C1—C2	1.4855 (19)	C7—H7	1.01 (2)
O2—C11	1.4600 (16)	C8—H8	0.938 (19)
C2—C3	1.3592 (18)	C9—N10	1.1501 (19)
C2—C9	1.4322 (18)	C11—C12	1.506 (2)
C3—C4	1.4350 (19)	C11—H9	0.969 (16)
C3—H3	0.964 (18)	C11—H10	0.976 (16)
C4—C8	1.3899 (19)	C12—H11	0.982 (18)
C4—S5	1.7323 (14)	C12—H12	1.00 (2)
S5—C6	1.7062 (15)	C12—H13	0.974 (19)
C6—C7	1.365 (2)		
			
O1—C1—O2	124.87 (13)	C6—C7—H7	124.0 (14)
O1—C1—C2	123.94 (12)	C8—C7—H7	123.2 (14)
O2—C1—C2	111.18 (11)	C4—C8—C7	112.58 (13)
C1—O2—C11	115.27 (10)	C4—C8—H8	123.9 (13)
C3—C2—C9	123.10 (13)	C7—C8—H8	123.5 (13)
C3—C2—C1	117.88 (12)	N10—C9—C2	178.99 (14)
C9—C2—C1	119.01 (11)	O2—C11—C12	111.16 (12)
C2—C3—C4	129.73 (13)	O2—C11—H9	107.1 (10)
C2—C3—H3	116.7 (11)	C12—C11—H9	113.1 (10)
C4—C3—H3	113.5 (11)	O2—C11—H10	103.1 (10)
C8—C4—C3	123.09 (13)	C12—C11—H10	111.3 (10)
C8—C4—S5	110.48 (11)	H9—C11—H10	110.5 (13)
C3—C4—S5	126.43 (10)	C11—C12—H11	110.2 (11)
C6—S5—C4	91.90 (7)	C11—C12—H12	107.6 (13)
C7—C6—S5	112.23 (11)	H11—C12—H12	111.4 (15)
C7—C6—H6	131.8 (13)	C11—C12—H13	111.0 (11)
S5—C6—H6	116.0 (13)	H11—C12—H13	107.7 (15)
C6—C7—C8	112.81 (12)	H12—C12—H13	109.0 (15)
			
O1—C1—O2—C11	1.03 (17)	C2—C3—C4—S5	0.03 (19)
C2—C1—O2—C11	−178.07 (10)	C8—C4—S5—C6	0.27 (9)
O1—C1—C2—C3	−2.45 (17)	C3—C4—S5—C6	179.99 (11)
O2—C1—C2—C3	176.65 (10)	C4—S5—C6—C7	−0.31 (10)
O1—C1—C2—C9	178.04 (11)	S5—C6—C7—C8	0.27 (14)
O2—C1—C2—C9	−2.85 (15)	C3—C4—C8—C7	−179.90 (11)
C9—C2—C3—C4	0.7 (2)	S5—C4—C8—C7	−0.17 (13)
C1—C2—C3—C4	−178.77 (11)	C6—C7—C8—C4	−0.06 (16)
C2—C3—C4—C8	179.71 (12)	C1—O2—C11—C12	−83.41 (13)

### Ethyl (E)-2-cyano-3-(thiophen-2-yl)acrylate (2) Hydrogen-bond geometry (Å, º)

**Table:**

*D*—H···*A*	*D*—H	H···*A*	*D*···*A*	*D*—H···*A*
C3—H3···O1	0.964 (19)	2.444 (19)	2.7998 (18)	101.5 (14)
C3—H3···O1^i^	0.964 (19)	2.45 (2)	3.3436 (18)	153.4 (15)
C6—H6···N10^ii^	0.99 (2)	2.49 (2)	3.465 (2)	169.1 (18)
C8—H8···O1^i^	0.94 (2)	2.50 (2)	3.3047 (19)	143.6 (17)

Symmetry codes: (i) −*x*+1, −*y*+1, −*z*+1; (ii) −*x*+1, −*y*+1, −*z*.

## References

[bb1] Al-Refai, M., Geyer, A., Marsch, M. & Ali, B. F. (2014). *J. Chem. Crystallogr.* **44**, 407–414.

[bb2] Al-Refai, M., Ibrahim, M. M., Geyer, A., Marsch, M. & Ali, B. F. (2016). *J. Chem. Crystallogr.* **46**, 331–340.

[bb3] Asiri, A. M., Al-Youbi, A. O., Alamry, K. A., Faidallah, H. M., Ng, S. W. & Tiekink, E. R. T. (2011). *Acta Cryst.* E**67**, o2315. 10.1107/S1600536811031941 PMC3200670 22058943

[bb4] Brenna, E., Crotti, M., Gatti, F. G., Monti, D., Parmeggiani, F., Powell, R. W. III, Santangelo, S. & Stewart, J. D. (2015). *Adv. Synth. Catal.* **357**, 1849–1860.

[bb5] Brenna, E., Gatti, F. G., Manfredi, A., Monti, D. & Parmeggiani, F. (2013). *Catal. Sci. Technol.* **3**, 1136–1146.

[bb6] Castro Agudelo, B., Cárdenas, J. C., Macías, M. A., Ochoa-Puentes, C. & Sierra, C. A. (2017). *Acta Cryst.* E**73**, 1287–1289. 10.1107/S2056989017010799 PMC5588562 28932456

[bb23] Crystal Impact (2014). *DIAMOND*. Crystal Impact GbR, Bonn, Germany.

[bb7] Faggi, E., Aguilera, J., Sáez, R., Pujol, F., Marquet, J., Hernando, J. & Sebastián, R. M. (2019). *Macromolecules*, **52**, 2329–2339.

[bb8] Groom, C. R., Bruno, I. J., Lightfoot, M. P. & Ward, S. C. (2016). *Acta Cryst.* B**72**, 171–179. 10.1107/S2052520616003954 PMC4822653 27048719

[bb9] He, J., Liu, Y., Gao, J. & Han, L. (2017). *Photochem. Photobiol. Sci.* **16**, 1049–1056. 10.1039/c6pp00410e 28497818

[bb10] Ibrahim, M. M., Al-Refai, M., Ali, B. F., Geyer, A., Harms, K. & Marsch, M. (2019). *IUCrData*, **4**, x191046.

[bb11] Kalkhambkar, R. G., Gayathri, D., Gupta, V. K., Kant, R. & Jeong, Y. T. (2012). *Acta Cryst.* E**68**, o1482. 10.1107/S1600536812016510 PMC3344592 22590354

[bb12] Kong, D., Li, M., Wang, R., Zi, G. & Hou, G. (2016). *Org. Biomol. Chem.* **14**, 1216–1220. 10.1039/c5ob02422f 26661067

[bb13] Liu, L., Song, J., Lu, H., Wang, H. & Bo, Z. (2016). *Polym. Chem.* **7**, 319–329.

[bb14] Liu, Q., Kong, F.-T., Okujima, T., Yamada, H., Dai, S.-Y., Uno, H., Ono, N., You, X.-Z. & Shen, Z. (2012). *Tetrahedron Lett.* **53**, 3264–3267.

[bb15] Miyazaki, E., Okanishi, T., Suzuki, Y., Ishine, N., Mori, H., Takimiya, K. & Harima, Y. (2011). *Bull. Chem. Soc. Jpn*, **84**, 459–465.

[bb16] Qian, S., Xie, Z., Liu, J., Li, M., Wang, S., Luo, N. & Wang, C. (2018). *J. Org. Chem.* **83**, 14768–14776. 10.1021/acs.joc.8b02325 30403868

[bb21] Sheldrick, G. M. (2015*a*). *Acta Cryst.* A**71**, 3–8.

[bb22] Sheldrick, G. M. (2015*b*). *Acta Cryst.* C**71**, 3–8.

[bb20] Stoe & Cie (2016). *X-AREA* and *LANA*. Stoe & Cie, Darmstadt, Germany.

[bb17] Xu, D., Li, Z., Peng, Y.-X., Geng, J., Qian, H.-F. & Huang, W. (2016). *Dyes Pigments*, **133**, 143–152.

[bb18] Yuvaraj, H., Gayathri, D., Kalkhambkar, R. G., Gupta, V. K. & Rajnikant (2011). *Acta Cryst.* E**67**, o2135. 10.1107/S1600536811028790 PMC3213575 22091152

[bb19] Zhou, N., Prabakaran, K., Lee, B., Chang, S. H., Harutyunyan, B., Guo, P., Butler, M. R., Timalsina, A., Bedzyk, M. J., Ratner, M. A., Vegiraju, S., Yau, S., Wu, C.-G., Chang, R. P. H., Facchetti, A., Chen, M.-C. & Marks, T. J. (2015). *J. Am. Chem. Soc.* **137**, 4414–4423. 10.1021/ja513254z 25768124

