# Supplementary material for: Synthesis, characterization, crystal structure and supra­molecularity of ethyl (E)-2-cyano-3-(3-methyl­thio­phen-2-yl)acrylate and a new polymorph of ethyl (E)-2-cyano-3-(thio­phen-2-yl)acrylate
Source: Acta Crystallogr E Crystallogr Commun. 2019 Aug 23;75(Pt 9):1357–61. doi: 10.1107/S2056989019011435 (PMC6727058; doi:10.1107/S2056989019011435)
Supplement: Supplementary file 6 [file e-75-01357-sup6.pdf]

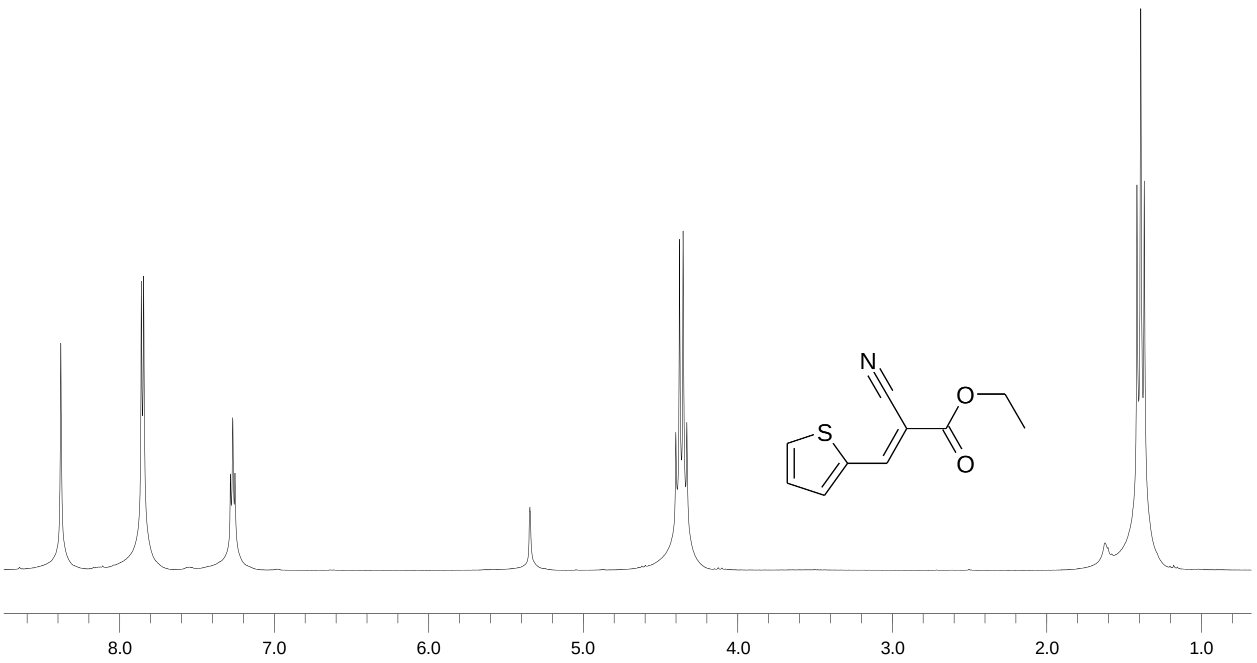

^{1}H NMR spectrum (CD_2Cl_2, 300 MHz) of (*E*)-ethyl-2-cyano-3-(thiophen-2-yl)acrylate (**2**)

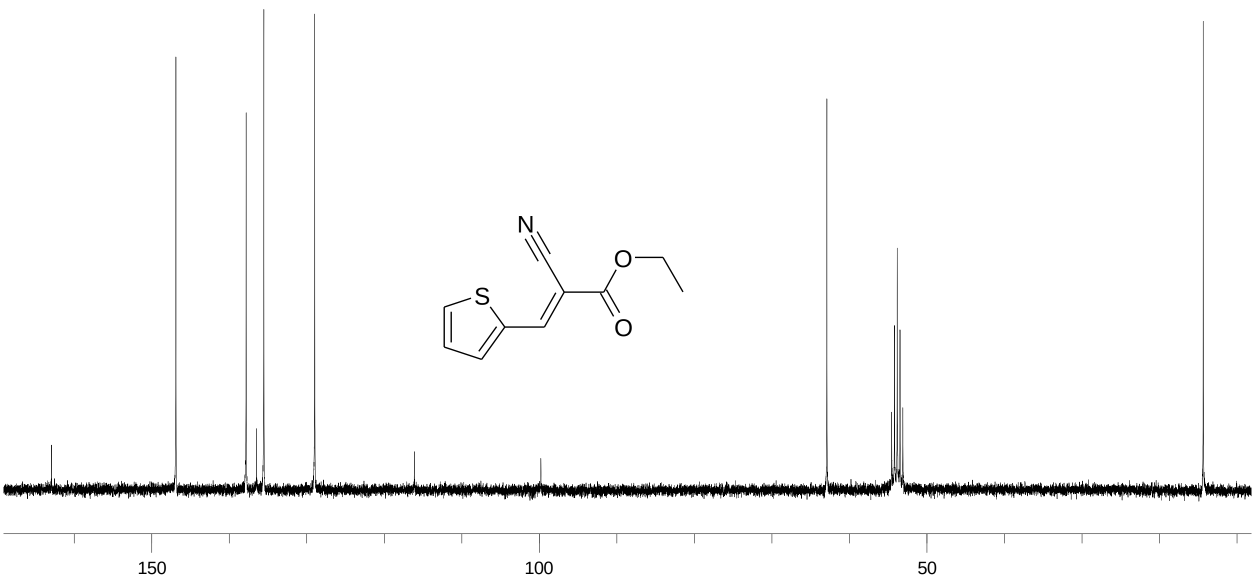

^{13}C NMR spectrum (CD_2Cl_2, 75MHz) of (*E*)-ethyl-2-cyano-3-(thiophen-2-yl)acrylate (**2**)

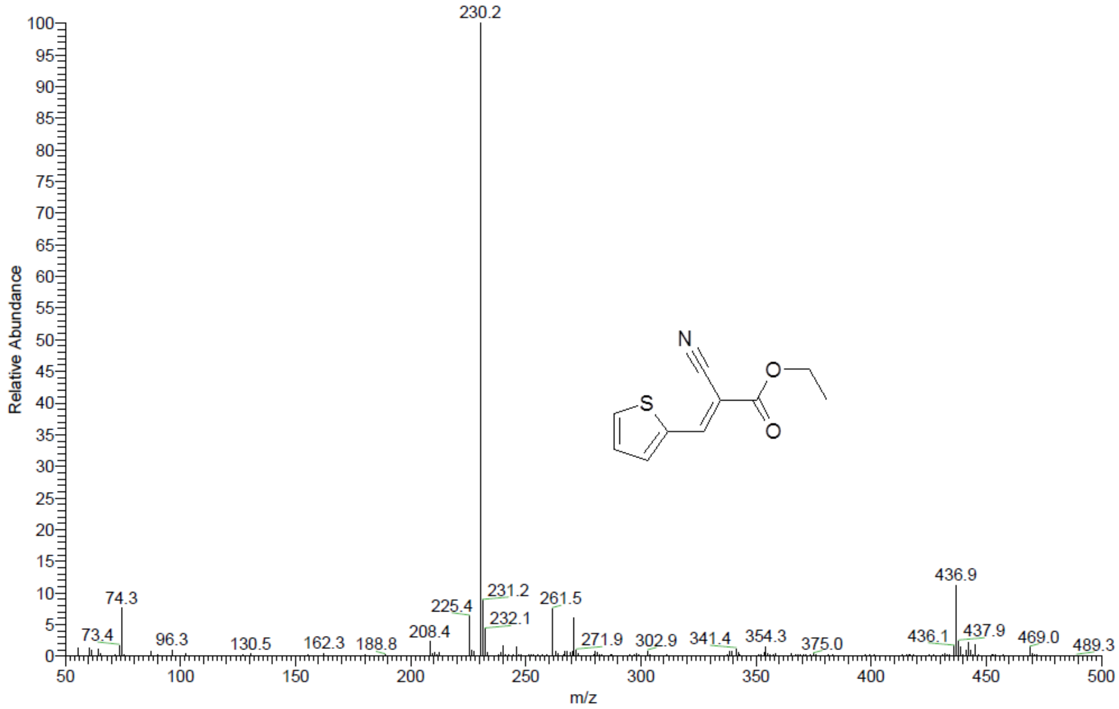

(+)-ESI mass spectrum of (*E*)-ethyl-2-cyano-3-(thiophen-2-yl)acrylate (**2**)

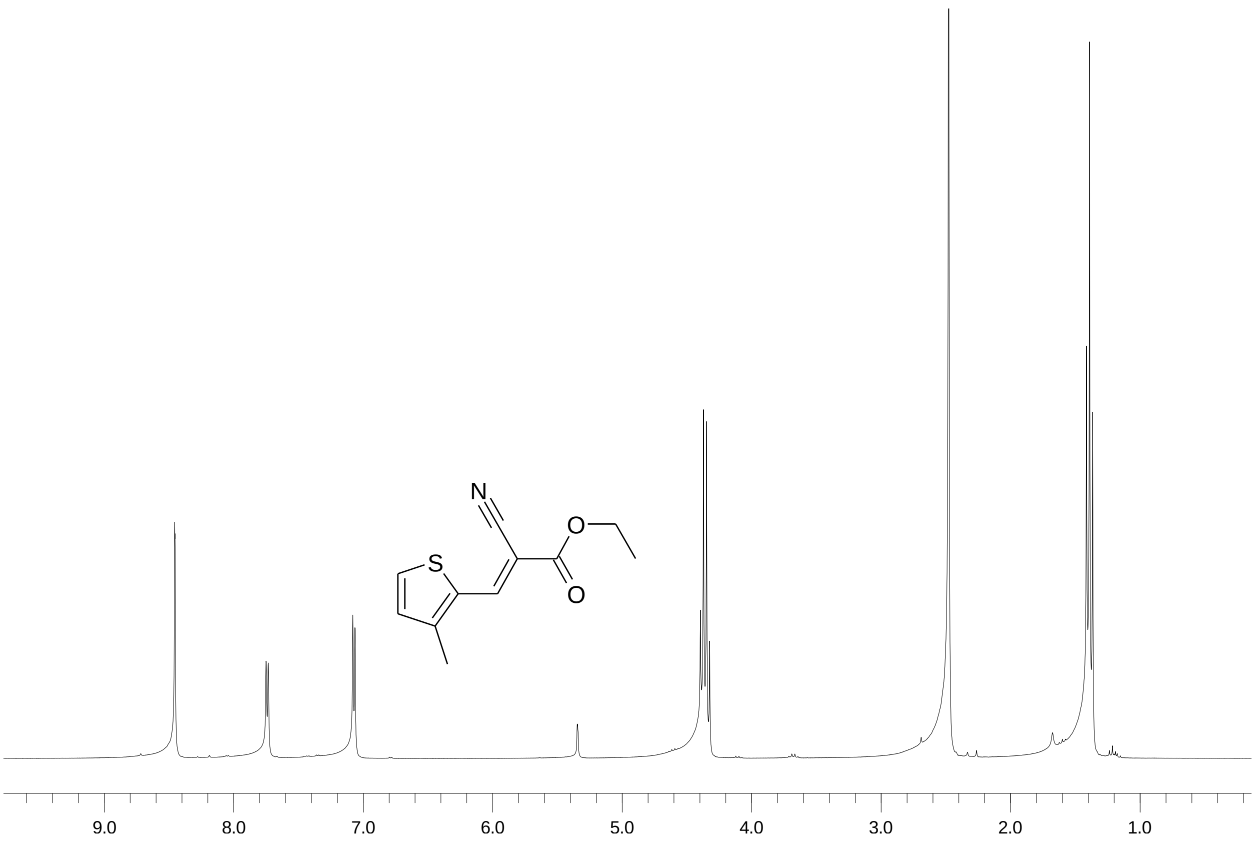

^{1}H NMR spectrum (CD_2Cl_2, 300 MHz) of (*E*)-ethyl-2-cyano-3-(3-methylthiophen-2-yl)acrylate (**1**)

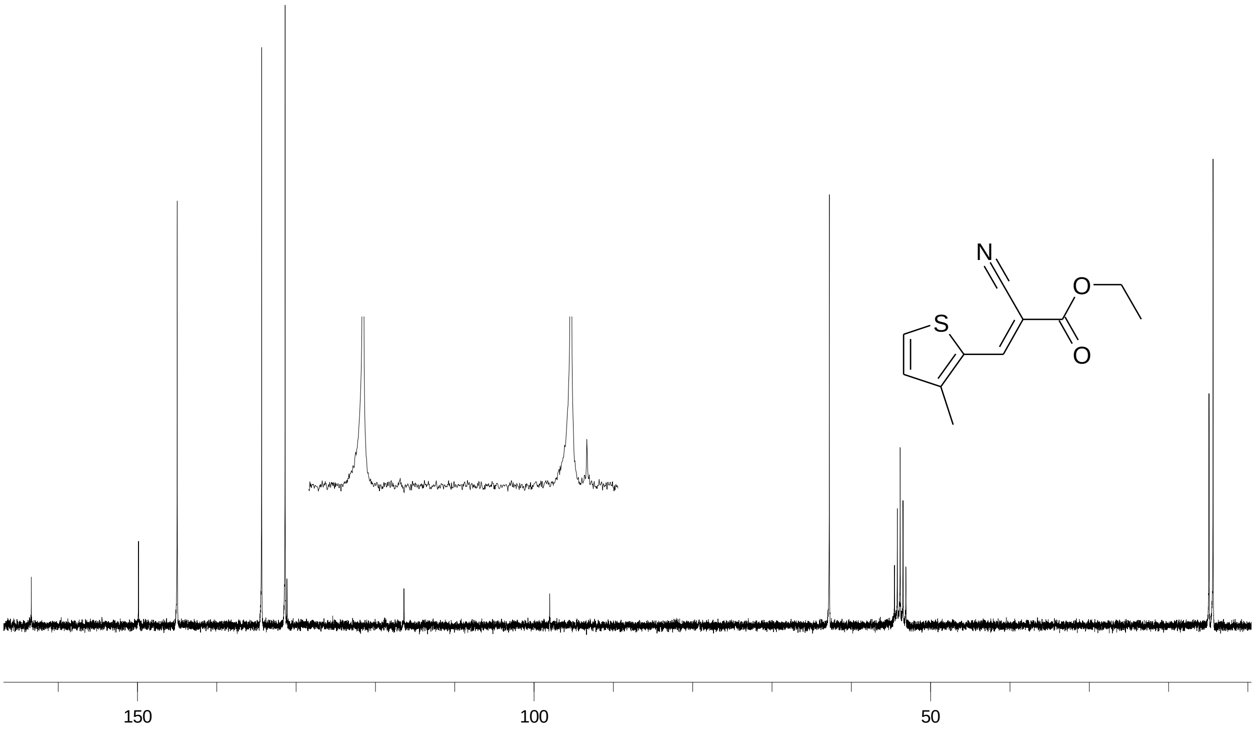

^{13}C NMR spectrum (CD_2Cl_2, 75 MHz) of (*E*)-ethyl-2-cyano-3-(3-methylthiophen-2-yl)acrylate (**1**)

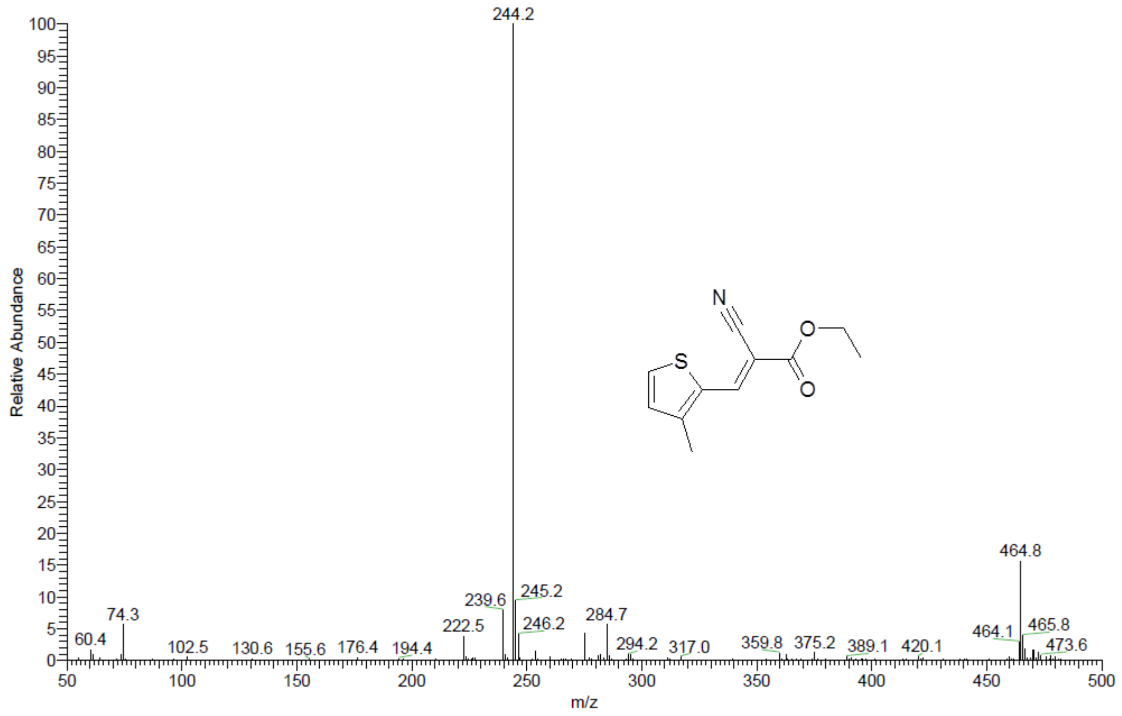

(+)-ESI mass spectrum of (*E*)-ethyl-2-cyano-3-(3-methylthiophen-2-yl)acrylate (**1**)
